# Serotypes and Patterns of Antibiotic Resistance in Strains Causing Invasive Pneumococcal Disease in Children Less than 5 Years of Age

**DOI:** 10.1371/journal.pone.0054254

**Published:** 2013-01-14

**Authors:** Chunfeng Liu, Xiaoyu Xiong, Wei Xu, Jimei Sun, Lijie Wang, Jiujun Li

**Affiliations:** 1 Department of Pediatrics, Shengjing hospital of China Medical University, Shenyang, Liaoning, China; 2 Department of Bacteriology, Shengjing hospital of China Medical University, Shenyang, Liaoning, China; University Medical Center Utrecht, The Netherlands

## Abstract

**Objective:**

The serotypes and patterns of antibiotic resistance of *Streptococcus pneumoniae (S. pneumoniae)* strains that cause invasive pneumococcal disease (IPD) in infants were analyzed to provide guidance for clinical disease prevention and treatment.

**Methods:**

The clinical features of confirmed IPD were evaluated in 61 patients, less than 5 years of age, who were admitted to our hospital between January 2009 and December 2011. The serotypes and antibiotic resistance of strains of *S.pneumoniae* were determined using the capsular swelling method and the E-test.

**Results:**

A total of 61 invasive strains were isolated. The serotype distribution of those isolates were 19A (41.0%), 14 (19.7%), 19F (11.5%), 23F (9.8%), 8 (4.9%), 9V (4.9%), 1 (3.3%), and 4, 6B, and 20 (each 1.6%). The percentage of *S. pneumoniae* strains resistant to erythromycin, clindamycin, and cotrimoxazole were 100%, 86.9%, and 100%, respectively. The percentage of *S. pneumoniae* strains resistant to penicillin, amoxicillin/clavulanic acid, cefuroxime, ceftriaxone, cefotaxime, cefepime, and meropenem were 42.6%, 18.0%, 82.0%, 18.0%, 13.1%, 13.1%, and 36.1%, respectively. The percentage of multidrug-resistant strains was 95.6%. Strains of all serotypes isolated in this study were highly resistant to erythromycin, cotrimoxazole, and clindamycin. Strains with serotype 19A had the highest rates of resistance.

**Conclusions:**

Serotype 19A strains were most frequently isolated from children with IPD treated in our hospital. The strains causing IPD are highly resistant to antibiotics.

## Introduction

*Streptococcus pneumoniae (S. pneumoniae)* is the predominant pathogen involved in bacterial infections and remains the leading cause of community-acquired respiratory infections in children. Pneumococcal disease is the leading cause of death worldwide among children less than 5 years of age [1]–[3] and endangers the health of Chinese children [4]–[7]. *S. pneumoniae* can result in bacteremia, meningitis, pericarditis, arthritis, and empyema; collectively these are referred to as invasive pneumococcal disease (IPD) [8] which can lead to severe illness, prolonged hospital stays, and death.

Currently, up to 13 serotypes are targeted in the available pneumococcal conjugate vaccines (PCVs). However, the formulations of these vaccines were initially designed to protect children against serotypes most commonly causing invasive disease in North America and may not reflect the serotype distribution across the world. Analysis of data from 6 ASEAN countries (defined as countries belonging to the Association of South East Asian Nations, ASEAN) showed that the most common disease causing serotypes of *S. pneumoniae* (in order of frequency) were 19F, 23F, 14, 6B, 1, 19A, and 3 [9] ^.^ Several studies showed the distribution of these serotypes to be diverse in various cities in China during different years [10], [11]. In one study, conducted from 2006 to 2008 in 11 hospitals in China [12], 171 isolates of IPD-associated *S. pneumoniae,* taken from children under the age of 14 years, were analyzed for serotyping and antibiotic resistance. It is worthwhile to note that analysis of local serotype distribution showed that the non-7-valent pneumococcal conjugate vaccine (PCV-7) serotype 19A was very common (14.3%–50% of isolates) in some northern cities. On the other hand, serotype 19A was rarely found (0%–10.7% of isolates) in southern and eastern cities; a result similar to those found in Southeast Asian countries [9]. PCV-7 covered more strains found in southern and eastern cities (70.4% of isolates) than in northern cities (36.7% of isolates). The reasons for the difference in isolate serotype among the different regions are not very clear. In September 2008, PCV-7 became available in the private sector in China, however the vaccine was not widely used either in the south or in the north, so vaccination with PCV-7 in those areas cannot explain the serotype difference. In addition, antibiotic resistance rates, although variable, have been shown to be increasing over time in China [7], [8], [10]–[12]. This has resulted in tremendous difficulties in the treatment of *S. pneumoniae* infections.

Thus, it is important to determine the local serotypes and antibiotic resistance patterns of *S. pneumoniae* isolates, from children with IPD, in order to provide evidence and guidance for the use of vaccines and antibiotics. The current study examines the serotypes and patterns of antibiotic resistance in *S. pneumoniae* isolated from patients less than 5 years of age, who were hospitalized for IPD at Shengjing Hospital of China Medical University in Shenyang, a city in North China.

## Methods

### 2.1 Patients

A total of 61 patients under 5 years of age, who were diagnosed with confirmed IPD between January 1, 2009 and December 31, 2011 in the Pediatrics Department, were studied. The diagnosis of IPD was based on cultures positive for *S. pneumoniae* that were taken from a normally sterile body site [13], such as blood, cerebrospinal fluid (CSF), or the pleural space. Forty-one strains were cultured from blood (60.3%), 20 from pleural effusion (29.4%), and 7 from the CSF (10.3%). The definition of pneumococcal meningitis was isolation of *S. pneumoniae* from the CSF or a clinical diagnosis of meningitis with pneumococcus isolated from another normally sterile site, such as blood. Among patients meeting these criteria, 7 had two isolates from different sites (7 isolates from blood, 4 isolates from pleural effusion, and 3 from CSF). In these 7 patients, only one isolate was included if both isolates expressed the same serotype.

This study was approved by the Medicine Ethics Committee of Shengjing Hospital affiliated with China Medical University (2011PS76K). Since this was a retrospective study, it was exempt from informed consent of the patients, parents, or guardians involved. Only *S. pneumoniae* strains preserved in the laboratory were used and no samples were taken directly from patients for this study; clinical data were collected in a manner that maintained patient privacy.

### 2.2 Isolation of bacterial strains

Blood samples were taken from children with IPD within 24 hours of admission. Cerebrospinal and pleural fluid samples were also taken from those children with clinical symptoms of sepsis, and/or meningitis or pleural effusion. *S. pneumoniae* isolates were analyzed and identified using standardized laboratory procedures, including colony morphology on blood agar, the optochin test, and the sodium deoxycholate solubility test. All of the isolates were confirmed as *S. pneumoniae* by testing for alpha hemolysis on blood agar, optochin susceptibility, and bile solubility (bioMérieux API system; France) and the Vitek 2 Compact system (bioMérieux) [14].

### 2.3 Serotyping of isolates

All isolates were serotyped by a capsule-quelling test using type-specific antisera (Statens Serum Institute, Copenhagen, Denmark). Typing was carried out by phase-contrast microscopy according to the published procedure [15].

### 2.4 Antimicrobial susceptibility testing

*In vitro* susceptibility tests were performed using Etest strips provided by the AB Biodisk Company (Solna, Sweden), and the minimum inhibitory concentrations (MICs) of 13 antimicrobial agents (penicillin, amoxicillin/clavulanic acid, cefotaxime, cefepime, ceftriaxone, cefuroxime, erythromycin, clindamycin, levofloxacin, cotrimoxazole, vancomycin, linezolid, and meropenem) were determined. Tests were performed following the recommended protocols of the United States Clinical and Laboratory Standard Institute (CLSI), and the 2008 amendments to the antibiotic susceptibility breakpoints for *S.pneumoniae* were adopted as criteria for determining drug resistance. The susceptibility medium was made using bioMérieux Mueller-Hinton agar. Multidrug-resistant *S. pneumoniae* isolates were defined as isolates that were resistant to 3 or more classes of antimicrobial agents.

### 2.5 Quality Control

The following strains were used for quality control: ATCC 49619 *S. pneumoniae*, ATCC 25923 *Staphylococcus aureus*, and *S. pneumoniae* standard strain 31001 serotype I (China Pharmaceutical and Biological Products).

### 2.6 Statistical Analysis

The data on antibiotic susceptibility and serotypes of the isolates were analyzed using SPSS version 17.0 (SPSS Inc., Chicago, IL) statistical software. The percentage of multidrug-resistant strains was calculated as the number of multidrug-resistant strains divided by total numbers of strains.

## Results

### 3.1 Clinical data

The 61 patients with IPD included 37 boys and 24 girls. The age of the patients ranged from 3 months to less than 5 years. Patients less than 2 years of age accounted for 37 cases (60.7%). Thirty-seven patients received antibiotics before admission, including macrolides (erythromycin and azithromycin) and β-lactam antibiotics (penicillin, amoxicillin/clavulanic acid, cefuroxime, ceftriaxone, cefotaxime, cefoperazone, ticarcillin, cefpimizole, aztreonam, and cefepime). Of the 37 patients, 21 received β-lactam antibiotics alone, 8 received macrolides alone, and 8 received a combination of antibiotics (β-lactam and macrolides). Thirteen had been given antibiotics for less than 3 days (1–3 days) and 20 for more than 3 days (median 7 days, range 4–20 days). The mean hospital stay was 21 days, and the maximum stay was 113 days. There were 50 cases of pneumonia, which included 28 cases complicated by empyema. There were 11 cases of pneumococcal meningitis; 7 CSF samples and 4 blood samples were culture positive for *S. pneumoniae*. Five patients, all under 2 years of age, died. Four patients died from septic shock and one patient died from brain dysfunction. Twelve patients received mechanical ventilation for periods ranging from 1 to 39 days.

### 3.2 Serotyping

A total of 68 strains were isolated from 61 patients with IPD. Among those strains, 7 strains that were obtained from the same patients had the same serotype; therefore, 61 strains were serotyped. Ten serotypes were identified, including 19A (25, 41.0%), 14 (12, 19.7%), 19F (7, 11.5%), 23F (6, 9.8%), 8 (3, 4.9%), 9V (3, 4.9%), 1 (2, 3.3%), and 4, 6B and 20 (1 each, 1.6%). The 7-valent PCV covers 45.9% of the serotypes, the 10-valent PCV (PCV-10) covers 52.5% of the serotypes, and the 13-valent PCV (PCV-13) covers 91.8% of the serotypes. A total of 37 strains were isolated from patients less than 2 years of age, including 15 of 19A (18/37), 6 of 14 (6/37), 6 of 23F (6/37), 4 of 19F (4/37), 2 each of 9V and 8 (2/37), and 1 each of 4 and 6B (1/37). PCV-7 and PCV-10 cover 54.1% of these strains and PCV-13 covers 85.4% of these strains.

### 3.3 Antimicrobial susceptibility

The susceptibility results are shown in Table 1. The percentage of *S. pneumoniae* strains resistant to erythromycin, clindamycin, and cotrimoxazole were 100%, 86.9%, and 100%, respectively. The percentage of *S. pneumoniae* strains resistant to penicillin, amoxicillin/clavulanic acid, cefuroxime, ceftriaxone, cefotaxime, cefepime, and meropenem were 42.6%, 18.0%, 82.0%, 18.0%, 13.1%, 13.1%, and 36.1%, respectively. None of the strains were resistant to vancomycin, linezolid, or levofloxacin. The percentage of multidrug-resistant strains was 95.6%.

**Table 1:** Antibiotic susceptibility of 61 invasive pneumococcal disease strains (minimum inhibitory concentration [MIC] method).

Antibiotics	% of isolates			MIC, mg/Ml		
	Susceptible	Intermediate	Resistant	50%	90%	Range
Penicillin	34.4	23.0	42.6	4	16	0.032–32
Amoxicillin/Clavulanic acid	50.8	31.1	18.1	3	8	0.025–32
Cefepime	39.3	47.5	13.2	4	17.6	0.064–32
Cefotaxime	57.4	29.5	13.1	1	4	0.016–16
Ceftriaxone	57.4	24.6	18.0	4	17.6	1–32
Cefuroxime	16.5	1.5	82.0	4	20.8	0.032–256
Erythromycin	0	0	100	>256	>256	2–>256
Trimoethoprim-sulfamethoxazole	0	0	100	32	1024	0.125–1024
Clindamycin	9.8	3.3	86.9	>256	>256	0.032–>256
Meropenem	14.8	49.2	36.0	0.5	1	0.008–2
Levofloxacin	100	0	0	1	1	0.25–2
Vancomycin	100	0	0	0.5	0.5	0.25–1
Linezolid	100	0	0	0.5	0.5	0.125–1

### 3.4 Correlation of serotype and drug resistance

As shown in Table 2, strains of all serotypes were highly resistant (with high MICs) to erythromycin, cotrimoxazole, and clindamycin, and no significant differences were detected between strains. Strains of some of the serotypes were also resistant to β-lactam antibiotics. Serotype 19A seemed to have a higher rate of resistance than other serotypes.

**Table 2:** Antibiotics resistance rates in different *Streptococcus pneumoniae* serotypes.

antibiotics	19A(25)		19F(7)		14(12)		23F(6)		Other serotypes(11)	
	No.	%	No.	%	No.	%	No.	%	No.	%
Penicillin	17	68.0	2	28.6	2	16.7	5	83.3	0	0
Amoxicillin/ Clavulanic acid	7	28.0	2	28.6	0	0	0	0	0	0
Cefepime	3	12.0	2	28.6	0	0	3	50	3	27.3
Cefotaxime	4	16.0	2	28.6	0	0	2	33.3	1	9.1
Ceftriaxone	3	12.0	4	57.1	1	8.3	1	16.7	1	9.1
Cefuroxime	24	96.0	6	85.7	11	91.7	5	83.3	2	18.2
Erythromycin	25	100	7	100	12	100	6	100	11	100
Trimoethoprim- sulfamethoxazole	25	100	7	100	6	50	5	83.3	8	72.7
Clindamycin	25	100	6	85.7	11	91.7	6	100	11	100
Meropenem	12	48.0	5	71.4	1	8.3	2	33.3	2	18.2
Levofloxacin	0	0	0	0	0	0	0	0	0	0
Vancomycin	0	0	0	0	0	0	0	0	0	0
Linezolid	0	0	0	0	0	0	0	0	0	0

Abbreviations: No., Number of isolates resistant to drug.

## Discussion

*S. pneumoniae* is a highly virulent organism and is associated with a high rate of mortality [1]–[3], [7]. Children, especially those below the age of 5, are particularly at risk from invasive infections. The data from our hospital [16] showed that approximately 74.4% of *S. pneumoniae* infections occurred in pediatric patients, and that pediatric patients accounted for 90% of invasive infections, with most occurring in patients less than 5 years of age.

Children under 2 years of age are known to be susceptible to IPD, and the mortality in these patients is high. In this study, patients less than 2 years of age accounted for 37 cases (60.7%) and 5 deaths, indicating that immature immunity among younger children may cause invasive infections to occur more easily. Consequently, special attention should be paid to young patients with *S. pneumoniae* infections. Compared with infections at other sites, pneumococcal meningitis is more severe and has a higher mortality rate, with a greater chance of adverse sequelae [17]. In this study, there were 3 deaths in patients with pneumococcal meningitis.

Based on the antigen profile of the polysaccharide capsule, *S. pneumoniae* can be divided into 46 serogroups, including more than 90 serotypes [18], [19]. However, according to worldwide surveys, almost 90% of invasive infections are caused by about 16 *S. pneumoniae* serotypes, including 19F, 23F, 19A, 6B, 14, and 6A. Serotyping provides excellent guidance for choosing a specific PCV. Several studies, conducted in various cities in China during different years, demonstrated great diversity in the distribution of serotypes of *S. pneumoniae* [10]–[12]. These results indicate that the serotypes of the IPD-associated *S. pneumoniae* strains from different regions are varied. In our study, IPD serotyping revealed that 19A was most frequently isolated from children with IPD treated in our hospital, accounting for 41% of cases. It might be the most common IPD pathogen in infants in Shenyang and the surrounding areas, followed by 14 and 19F. The dominance of serotype 19A in Shenyang is similar to the recent trend seen in the Beijing area [5], [12], possibly due to the close proximity of these 2 regions. The diversity of *S. pneumoniae* serotypes from different regions and differences in the serotypes that are covered by the various PCVs provide guidance for the selection of the appropriate vaccine in different regions. Our results indicated that PCV-7 would cover 45.9%, PCV-10 would cover 52.5%, and PCV-13 would cover 91.8% of the serotypes found in Shenyang. For vaccination and prevention, the major target population consists of children under the age of 2 years. PCV-7 and PCV-10 would cover 54.1% of this population and PCV-13 would cover 85.4% suggesting that PCV-13 should be the major target for future development and applications in the Shenyang areas. The spread of type 19A in western countries was found after the large-scale use of PCV-7, because 19A is not covered by PCV-7. However in North China, serotype 19A became prevalent in IPD before the introduction of PCV-7, and a similar phenomenon was observed in South Korea [20]. Our findings also indicated that serotype 19A was still one of the most common serotypes in Shenyang and was not related to the introduction of PCV-7, a vaccine not widely used in this area. The spread of highly resistant strains of serotype 19A reported in our study suggests that the widespread use of antimicrobials may provide the selective advantage necessary for resistant serotype expansion, similar to the situation in Korea [20].

*S. pneumoniae* resistance to antibiotics is a growing problem and has become an important prognostic factor, since it is directly associated with persistent disease or disease mortality [21], [22]. In this study, most of the 61 patients with IPD were treated for a period of time before being admitted to our hospital and were admitted because of exacerbation of disease. Our susceptibility analysis revealed that resistance to *S. pneumoniae* is a serious problem. The rate of multidrug resistance was 95.6%, and the rate of resistance to macrolides and clindamycin was as high as 100%. In addition, the rates of resistance and insensitivity to penicillins and cephalosporins showed an upward trend. This indicates that *S. pneumoniae* resistance to antibiotics plays a large role in the poor efficacy of antibiotics in both the inpatient and outpatient settings. Because most patients in our study had been treated before admission to the hospital, an unknown fraction of strains analyzed in the study could originate from IPDs that failed to respond to treatment. Thus, the strains tested could have been enriched for resistant isolates. Therefore, the high rates of antibiotic resistance among pneumococcal strains in our study may not completely represent resistance of pneumococcal strains circulating in the population of this region.

Studies have shown that *S. pneumoniae* strains resistant to penicillin and multidrug-resistant strains had significant correlations with specific serotypes [18]–[20]. Some of the multidrug-resistant serotypes may be disseminated worldwide. In this study, the rates of multidrug resistance of serotypes 19A, 19F, 14, 23F, 1, 4, and 6B were 100%. Generally, serotype 19A seemed to have a higher resistance rate than other serotypes, although no significant difference was found in most instances due to the low number of isolates in our study. Serotype 19A is not a serotype that is covered by the 7-valent vaccine, which should be of concern. Additional studies on the molecular mechanisms of resistance as well as on establishing the clonality of the resistant isolates are needed.

Studies in many countries have shown that use of PCV decreased the overall incidence of IPD among children [23]–[26]. The increase in cases of IPD among infants and toddlers and the increasing drug resistance of invasive *S. pneumoniae* also suggest the need for the implementation of vaccination in infants and young children. Because the non PCV-7 serotype is increasing in prevalence, broader serotype coverage such as PCV-13 might be optimal for future vaccination in Shenyang.
